# Between Presence and Commitment: A Qualitative Exploratory Study of People with Visual Impairment in Polish Religious Communities

**DOI:** 10.1007/s10943-022-01633-2

**Published:** 2022-08-24

**Authors:** Magdalena Maciejewska

**Affiliations:** Nicolaus Copernicus University, Toruń, Poland

**Keywords:** Blind people, Community, Christianity, Presence, Commitment

## Abstract

This article aims to identify factors that may be important in the inclusion process of people with disabilities in religious communities. This text was based upon the interviews conducted with 10 respondents who belonged to Christian communities. They were characterised by a diverse approach, and are therefore referred to in this article as spiritual settlers, spiritual pilgrims and spiritual wanderers. These were then associated with theoretical terms such as presence, affiliation and commitment, to analyse the procedures of the respondents' self-reported functioning in these religious communities.

## Introduction

People, regardless of personal factors such as their state of health or level of fitness, generally seek meaning in their lives (Głaz, 2006). Finding such meaning, in the opinion of Frankl (2014), who was both a psychiatrist and a prisoner of the Auschwitz concentration camp, is a basic human need. However, its discovery is not easy, as a person’s surroundings tend to drive them to focus on the rapid disclosure of new needs, which must be taken care of as soon as possible; this particularly upsets the existential balance of people who find themselves in a society where the following features are valued:

the pursuit of success; those who enjoy success and happiness deserve universal adoration (...) The value of all others who do not meet the above criteria is virtually ignored – thus blurring the crucial difference between being a worthful human being in terms of dignity and being worthful in terms of utility (Frankl, 2014, p. 219).

This may prove to be particularly important for people experiencing disability, as their possible range of activities may change over time, due to variance in physical limitations. They may thus spend a great deal of effort trying to understand the reasons for this and seeking to redefine their places in society, as without such redefinition, it may be difficult for people whose lives are forcibly different from the expected norms to function in an externally unchanged reality.

Many of those experiencing disability seek a reference point for their lives and the activities they undertake daily in the religious sphere. It includes both the aspect of the relationship with God and with the community. Because of religious doctrines that include the issue of special care for weak, sick and disabled people, this seeking is linked to the expectation that in religious communities, people with disabilities will experience a greater level of acceptance and understanding of their weaknesses. In terms of discovering the meaning of a forcibly modified life, based on empirical attempts to find one's place in the new experiential reality, having a religious community can help, as this thus becomes a fragment that the person entering such a community can become responsible for (Horowski, 2019).

In the view of the last 15 years, there has been a lot of research on the religious functioning of people with disabilities. For the most part, these research studies dealt with the aspect of disabled people's relationship with God and their religious practices. For instance, participation in religious services has been found to positively influence individuals' sense of meaning in life (Koenig et al., 2012; Krause & Hayward, 2012). The relationship between the sense of meaning in life, satisfaction and well-being of people with disabilities and religious practices has been highlighted (Chlan et al., 2011; Janocha, 2011; Johnstone et al., 2008; Marini & Glover-Gra, 2011), also considering the role of religiosity in the process of adjustment to disability (Brennan & MacMillan, 2008; Campbell et al., 2010; Giaquinto et al., 2007; Glover-Graf et al., 2007; Johnstone et al., 2007; Marini & Glover-Gra, 2011; Waldorn-Perrine et al., 2011; Yampolsky et al., 2008). An increasingly common research topic related to the religiosity of people with disabilities is their accessibility to the church and the church’s adaptation to the abilities of people with special needs (Borowska-Beszta, 2021; Carter et al., 2022).

About people with visual impairment, research on selected aspects of their religiosity and spirituality, such as the relationship between spirituality and religiosity and rehabilitation has been emerging recently (Brennan & MacMillan, 2008). In addition, religious strategies for coping with a disability (Abarghouei et al., 2017; Brennan et al., 2001) and the importance of extraordinary pastoral care in the context of social inclusion (Lipiec, 2011) have been explored. In Poland, Szabała (2021) attempted to characterise the religiosity of people struggling with visual impairment and reached similar conclusions to Janocha (2011), who claimed that the more advanced the degree of visual impairment, the higher the level of religiosity, and Domagała-Zyśk (2017) draws attention to the issues related to the Christian context of understanding disability.

Hence, it is not difficult to notice that the research carried out primarily concerns with the relationship between God and the people with visual impairments, their religious practices or pastoral care. Little to no attention is paid to the very functioning of people with visual impairment in religious communities. This is a particularly important issue because for these people to develop religiously, they should also have a place for themselves in religious communities. Since the functioning of people with visual impairment in the religious communities they belong to and their attitude towards the communities are unexplored phenomena, the research described in this article focused on these aspects. Moreover, when talking about functioning in the communities, it is important to remember that it depends not only on the other members of the community, but, above all, also on the people concerned. The omission of this thread from the research may wrongly suggest that the degree of engagement is solely dependent on the openness of the community.

This research is an attempt to find the answer to the following question: What is the nature of involvement of persons with visual impairment in the religious community? Thus, an attempt is made to identify factors that may be important in the process of including people with disabilities in religious communities. The article is structured as follows: the first part (preceded by an introduction) presents the most important issues related to the presence and functioning of people with disabilities in the community; the second part presents the methodological aspects of the research: and, the third part analyses the results of empirical research results.

### People with Disabilities in Community

The definition of community reflected in this process has caused difficulties for researchers for centuries, becoming the cause of disputes among sociologists of various backgrounds (Mikołajewska, 1999). Tönnies (2012), the father of the theory of community, considered the foundation of all communities to be the community of blood, which was both natural and characterised by the greatest degree of permanence. Cohabitation was then seen as the main foundation for the creation of a community of spirit (Tönnies, 2012). Such communities, which remain most closely associated with the idea of religious community relevant to the present work, take what a German sociologist might define as a common approach to worship. The category of community can also be considered in relation to the institutional-social dimension, as done by von Hildebrand (1930), who understood it to be any collection of persons who are, in some real and objective way, joined together and can thus be defined as a new social unit. This definition, however, despite its broader social dimension, remains far removed from the relationality that is generally deemed important in a religious community. Cudowska (2009), however, produced a definition that, due to its personalistic character, offers a better point of reference for current considerations, claiming that.

the essence of community is the sentiments held for other people, the world and oneself. Community is a gradual phenomenon; its strength is determined by the level to which its members experience a sense of solidarity and relevance. In the reality dominated by individualisation, a human being entangled in a network of global connections and dependencies loses the possibility of “rooting” and looks for a community that would be a “safe harbour” in the everyday effort of becoming (p. 211).

Causal action that requires people to be involved does not always exist within the context of a social group. However, as aspects such as family life, social relationships, and work tend to involve numerous links of demonstrated agency with other people, it is important to consider whether mere presence in a community signals at least some commitment or readiness to engage. This makes it necessary to acknowledge the distinctness between the terms “presence” and “commitment”, which many people overlook because they consider places to *be* in a literal sense, in terms of being located in a particular space. The existence of the word *sojourn*, which is used to signal a certain longevity of *residence* rather than a purely temporary *visit*, but which falls short of permanence, can make semantic unambiguity difficult, however, as is clearly also possible to *be* in isolation from any set of geographical coordinates in terms of identity. In the case of a religious community, such *be*ing can refer to a spiritual connection or a sense of belonging to a particular group despite an inability to coexist physically with it. Belonging, nevertheless, cannot be assumed to only refer to the commonplace of *be*ing in a given place in spirit rather than in body: it can also refer to identity declarations that are not linked in any way to concrete practices. These ontological considerations and complexities prompt reflection on the differentiation of several terms relevant to this work, particularly “presence”, “belonging”, and “commitment”. The feeling of those who are merely present at a place differ from the people who feel a sense of belonging, and they further differ from those who engage in community activities. Perception of the common good is crucial in this case, that is:

such a good that is a genuine good both for the community as a whole and for each individual living in that community (...). The good can thus be called such a real object of human action that can become a real goal of every personal endeavour. And in this sense, such a real goal (the good as currently desired) can be an analogically common goal for all persons living in that society (Krąpiec, 2001, pp. 629–630).

The implementation of the common good is not simply the responsibility of a leader nor elected representatives, but rather of the whole community. Recognising this is the first step on the road to commitment, which is manifest in the co-determination of those responsibilities or initiatives that can be considered to be of benefit to each individual and to the population under consideration. However, the desire to co-produce a common good is not often the main reason for involvement in a community. Instead, through community, people are able to find a secure place and some form of stability, whether social, spiritual or otherwise, to help them realise their humanity and to discover and reflectively live out their own freedoms and subjective worth, based on taking responsibility for the functioning of the group, which, to some extent, reflects the person’s aims or views. This, in turn, relates to one of the basic human needs, which is the need for belonging, and thus offers the main reason for the creation of communities, (Zaffalon-Peter et al., 2015). This need is related to the desire for non-self-identification with ideas, based on a need for rootedness in the individual that facilitates the discovery of identity (Ryan & Deci, 2004). Thus, simply *being* part of a community, with the associated identification or lack thereof, enables a person to discover their identity; however, the many discrepancies among group members mean that this may not be confirmed in practice, leading to the abandonment of commitment and belonging, or to the person leaving the group altogether. Nevertheless, in cases where a sense of internal and external coherence is achieved, this promotes engagement and further exploration alongside verification of identity. The sense of acceptance as well as a certain level of safety and security are the necessary to achieve this state. As Gomes (2002) pointed out: “A person needs to feel at home in order to maintain mental health, in other words, needs to belong to something, to recognise and be recognised, to identify himself and be identified by his peers, to have some connection and be part of a larger whole that welcomes and protects him” (p. 36).

This not only suggests that home, peer, and religious communities meet similar needs, but also, more importantly, draws attention to the perspective underpinning the present discussion, which is based on the ideas of presence and commitment. One can belong to a home community, identifying as part of a given family, based on surname or other relationships, and it is also possible to become involved in family matters by actively participating in events and experiencing the common joys and worries of the family, fulfilling the duties resulting from an assigned role and initiating various activities spontaneously. Similar possibilities apply to other communities, even where these lack a formal structure and are characterised by different catalogues of rights and duties, as long as these allow people to be present (in terms of a temporary, non-committal stay), to belong (a level of commitment to certain spheres and for a certain period of time), or to be committed (taking up relatively permanent residence). It is possible, therefore, to be present without moving on to belonging and commitment, or to belong without making a commitment; however, it is impossible to be committed without belonging and without some form of presence, which may be defined not only as a *sojourn* in a given place, but also as being present *in spirit*; this is necessary, as activity for the common good requires at least a mental identification with a given group.

Despite the diverse functionality of individual members of a community, their equality in terms of the dignity of the person should be assured, and they thus should all have the opportunity to participate in promoting the common good. This is one of the important markers of a united community, and the presence of sick, elderly or disabled people in the community should have a positive impact with regard to others appreciating the value of health and fitness: “through their presence in the public space they show that it is possible to do ordinary things, but also quite difficult ones, without being fully fit. Through their sensitivity they help to see the reality of the world as it is, not as we would like to see it” (Duda, 2014, p. 285).

Contact with community members struggling with such difficulties is often a reason for others to reflect on the source of their strengths, optimism, and persistence. This in turn can lead to a profound transformation of attitudes. The first feeling often experienced by people meeting with a person with any disability may be a negative one, related to the discomfort of the interruption in human consciousness of a stream of information of an incidental, typical, and predictable nature. By forcing consideration of the ultimate categories of human life, questions about the meaning of suffering and the essence of experiencing certain situations are raised in the human mind.

Coming into contact with the disabled causes shock precisely by evoking this “unwanted” context. The sense of disability and the experience associated with it evoke different human reactions: from compassion and pity, through dismay and fear, to outright, open rejection and resentment. However, these will always be reactions that testify to the “extraordinary” nature of the experience, to the disruption of the ordinary, everyday course of the stream of consciousness. The experience induced by eye contact with disability belongs to the sphere where intuitions and emotions connected with thoughts about one's own illness, about the threat of illness and death of people close to us are located. In other words, it evokes our horizon of contingency (Chudy, 2001, p. 79).

Thus, it can be concluded that the experiences that people with disabilities bring to the religious community are not related to the meaningfulness of particular dimensions of the present culture, but instead encourage reflection on various aspects, among which is the potentiality of existence.

Every human being is potential – unfinished – and develops its potentialities from conception until death. We differ as to the extent of these potentialities and their development, but both those with a narrow and broad range of achievements are seen in personalism as equal to their human dignity and right to be respected. Every human being is an adventurous being, experiencing every day his or her fragility in the face of fate and nature. A fully healthy and strong person can become weak and disabled one minute after a car accident (Domagała-Zyśk, 2008, p. 191).

Community activities are therefore verifiers of the attitudes of other members of the community in a manner that can be described as serving as a moral test of a given human community (Chudy, 2001).

## Methods

The empirical material presented in the next section of this article is drawn from research conducted in 2020 on the subject of spiritual development among persons with visual impairment in relation to their self-selected religious communities. The approval was granted by the Research Ethics Committee by the Faculty of Philosophy and Social Sciences of Nicolaus Copernicus University (Application No: 2/2022/FT). This article is based on that part of the respondents’ replies in which they referred to the nature of the involvement of persons with visual impairment in the religious community as highlighted in the research question. This research was conducted using a qualitative strategy based on examining individual cases, with data gathered via in-depth interviews based on previously prepared interview outlines. The interview focus was thus with concerned four main areas: relationships with God, interpersonal relationships, relationships with oneself, and relationships with elements of the external world not mentioned previously. The application of this technique and the appropriate tools allowed the interview conversations to include several particularly sensitive issues, including those related to religious experiences and participants’ personal backgrounds. However, due to the fact that vision loss is most often a traumatic event, issues specifically related to this were not investigated.

Ten of the respondents struggled with visual impairments, identifying as blind, persons with visual impairment, or unsighted; they also belonged to relevant Facebook groups, “Niewidomi i Niedowidzący – Bądźmy razem” and “Osoby niewidome i słabowidzące”. The main criterion for selection was membership (whether at the time of the interview or at any time previously) of a Christian religious community. Religious communities that are attached to parishes and religious orders are very popular in Poland. Despite the ongoing global secularisation processes, in 2018, the number of parish-based Catholic Church organisations, which often have a local character, was 65,500. Their formal members were 2.57 million people (ISKK, 2020). However, it is difficult to determine the number of religious communities that survived the pandemic in Poland and the number of people, including those with visual impairments, who currently belong to these communities. The analysed respondents included Catholics and Protestants, 9 laypersons and 1 consecrated person. Religious differences, however, were not essential to the respondents’ attitudes towards religious communities. These issues were addressed in subsequent sections of the article. All interviews were conducted via telephone due to the pandemic situation at the time of the research, and respondents' consents were thus recorded and transcribed afterwards, along with the interviews themselves. The interviews ranged in length from 45 to 90 min. To prevent the personal identification of individuals, all names have been changed. Information on the age, education, religion and religious community of the respondents are presented in Table 1.

**Table 1:** Demographic characteristics of the respondents. Source: own work

Code	Age	Education	Religion	Community
A1	24	Higher education	Catholic	Catholic Charismatic Renewal “Gałąź Sykomory”
A2	27	Higher education	Baptist	Baptist Christian Church “Droga Zbawienia”
A3	28	Higher education	Catholic	Catholic Charismatic Renewal in Laski, Catholic Charismatic Renewal “Kyrios”
K1	45	Higher education	Catholic	Catholic Charismatic Renewal, International Association Faith and Light, School of New Evangelization
K2	28	Higher education	Catholic	Academic Evangelizing Community „Woda Życia”, Community of People with Disabilities in the Church of St. Dominic
L1	54	Vocational education	Catholic	Prayer Community in the Church of St. Dominik
S1	60	Basic education	Pentecostal	Pentecostal Church of Christ the Savior
M1	49	Higher education	Catholic	Catholic Charismatic Renewal
R1	37	Middle education	Catholic	Oasis of the Young People in Krościenko, “Lednica 2000”
S2	50	Middle education	Catholic	Association of the Catholic Apostolate

## Results

After analysing the ten in-depth interviews, it evident that the respondents respond to religious communities differently. In this text, the respondents were divided into three categories: spiritual settlers (1), spiritual pilgrims (2) and spiritual wanderers (3). The characteristics of these categories are presented in Table 2.

**Table 2:** Attitudes of people with visual impairment towards religious communities*.* Source: own work

Category	Description
Spiritual settlers	Engaged in the life of the religious community and ready to take on new challenges in order to develop themselves, as well as the community. They establish long-lasting relationships with other members and feel strongly engaged in the community
Spiritual pilgrims	Seek a place for themselves in different religious communities. They have a set goal, which may be modified during their search. They attempt to subjectively care for the religious group and its members
Spiritual wanderers	They often change religious communities. Sometimes they use them only when needed for some reason. They do not become permanently engaged with a particular religious group

### Spiritual Settlers

Representatives of the former were referred to as spiritual settlers. In the case of the first group persons, their relationship with God and the religious community is already formed, and therefore neither their presence nor sense of belonging is sufficient for them. They are committed to their community, which suggests, among other things, an ability to indicate precisely the scope in which their activities contribute to its development. It is not uncommon for such individuals to decide to take on new challenges in spite of the fear associated with change, however, including, but not limited to, making contact with people who question topics of faith. An interesting attitude to this issue was presented by S1, who argued that.

[saying] spiritual conversations is too much ... It is more of a general human thing. Because you cannot have your head too high in the sky either, but you also have to walk on the ground, the reality of life. You know what you can say to the hungry about the Lord God. That is why you would not say “oh, look how beautiful” to a man who has nothing to eat. That is why we are not just talking about spiritual, but life, you have to live normally, function normally. (…). The most important thing – to be human to another without looking at who is who.

The active element of commitment was also highlighted by A2, who runs the Sunday school. She said.

I believe very much in everything that is written in the Bible and even in the running of this school, because it is written to go into all the world and preach the gospel to every creature. Well, actually we do that at school, because we read the Bible with the children, we talk, we talk about the Lord God, about Jesus Christ, and you can say that we fulfil the verse that is written in the Bible.

It is worth noting that for some respondents, as in the case of A2, a relationship of deep faith that drives community commitment is evident, while in others, such faith is not so strongly emphasised. A willingness to take on new challenges of a completely different kind was also demonstrated by S2, whose involvement focused on going beyond herself and undertaking unknown activities, driven by a desire to be involved and engaged with the community:

I liked to wash the dishes when the old chapel was there, because the new one now has a dishwasher. Although, when it was a birthday or something, the dishwasher couldn't cope. But at first I had this thing about not being fit to push myself there. A group of able-bodied people there... Let me put it another way – I feel bad when there's something new, new furniture, a dishwasher or all that. I don’t have luxuries, we live modestly at home, and when something new comes along, I wait and watch at first so as not to spoil anything, but I’m a slow learner, just like with this dishwasher, which means I'm changing. We learn for the rest of our lives.

It was also evident from the respondents’ statements that the need for security discussed in the theoretical section of this work was very important with regard to their commitment to community activities. Where such needs are left unsatisfied or where people feel unwanted, distancing from joint initiatives occurs. In the case of A2, however, who has been able to manage her shyness and to develop greater empathy through her commitment to a religious community, her involvement has led to improvements in her other relationships and to greater social engagement:

I think that with this empathy I have a kind of ability, especially among people with disabilities, to lean on these people, to listen (...). I also used to interact a lot with people after divorce and my mother used to laugh at me that I was confessional. As they talked to me, I tried to keep their spirits up somehow and I even succeeded (...). It is this empathy that manifests itself all the time.

The statements of these spiritual settlers clearly indicate that the community is treated by them in a subjective manner: despite the fact that its members are not connected by blood ties, they treat the community as a family. This is facilitated, among other things, by the regular organisation of meetings that are not inherently religious in nature but which focus on integration and entertainment elements, as pointed out by A2:

during the week there are also various meetings, either prayer meetings or groups where people meet in their homes and discuss various biblical themes (...) Meetings are held in several homes. They decide who is going to be the leader, because there is a tradition in this church that before the group starts, before the meditation on God’s Word and the topic, there is a snack in the form of pizza, soup or some sweets, so it starts with a common feast, followed by the introduction of the topic. There are also various questions that the participants of this group can ask or tell, and it ends with a prayer.

The skilful blending of the sacred with the secular is thus turns extremely important, which may prompt reflection on the community of dwelling as preceding the community of spirit in the manner Tönnies suggested (Tönnies, 2012). In this case, while no such dwelling takes place, certain elements of this process, such as sharing meals or leisure time, which are not directly related to spiritual matters, act the glue that binds participants to their communities.

### Spiritual Pilgrims

Spiritual pilgrims are characterised by a particular propensity for exploration, which involves travelling through different religious communities. This is not an aimless wandering, however, as it is undertaken in the belief that, at the end, there will be a place where the person will want to commit to the common good as well as experiencing their own needs being met. During the pilgrimage process, however, this goal is subject to modifications connected to, among other things, the discernment of vocation. A3, among others, mentioned this need:

everyone is called to something, and I understand it this way, that everyone lives for something. In Laski (village in Poland) I began to understand this. Maybe I have become more confirmed in my vocation because, for example, I have felt for some time that I am called to live in marriage. I feel it, I have a boyfriend, I don't know what will come of it, but I feel it. And for some time now more and more and maybe that has changed.

A growing awareness of destination, which is likely to have been unknown from the very beginning, was highlighted in the spiritual statements of those identified as pilgrims. Phrases suggesting trajectories related to the process of exploration appeared prominently in the statements of this group, and some, such as A1, saw the community as simply a supportive place in which to establish a relationship with God and with oneself:

since these musical meetings were rare, I needed something permanent, something that would strengthen me in my faith, people to support me and some guidance on how to establish relationships with God and myself. I think that's why I went there, and it was like: we'll see... Maybe not with any particular aim, but we will see how it goes. (...) At first it was nothing regular, but then it became more and more frequent, more often, until finally every week.

The effort put into the search process and the awareness of purpose in this case not only led to a shift from presence to commitment, but also to a recognition of the subjectivity of the community. Better knowledge of oneself, which often emerges from such processes, can benefit the community; however, it can also lead to its abandonment, in a move characteristic of the spiritual pilgrim. A1 highlighted this further:

I feel that it is not mine, so I think slowly, because it is going very laboriously, I am searching, but it ends up speaking actually. I would like to find something with my peers, some pastoral ministry at university, and if not at university it might be somewhere else, and it might turn out that suddenly these meetings with peers are not suitable for me either, because maybe I won't be understood there too much. It may be that people are afraid of me as a disabled person, that this will be insurmountable. I may eventually find myself returning to the Gałąź Sykamory.

In many cases, spiritual pilgrimage also involves actual pilgrimage, to shrines and places considered special by the followers of a particular religion. Wuthnow's types of spirituality suggest that non-attachment to a place traditionally associated with the sacred can be conceived in a slightly different way in such cases, and miraculous springs, places of revelation located in nature, or previously non-religious sites played an important role for many of the persons with visual impairment interviewed who were otherwise involved in religious communities (Wuthnow, 2003). These sites became places for increased reflection and intense experiences which, due to their connection with the emotional sphere, affected the respondents’ experience of God's presence. This act of seeking spirituality can therefore be seen to more sacred for them than it at first appears, based on them immersing themselves in a sphere that could be seen as more predominantly secular. Pilgrimage, however, was not a marker unequivocally attributed solely to this attitude: such activities, most often stemming from a desire to regain sight among respondents in this research, appeared in all groups. M1, among others, mentioned attending masses with prayer for healing, and she discussed the emotions involved:

I was a member of a community for about 10 years, and I got there through friends. Later, when I began to lose my sight, I somehow became more involved with the community. There I took part in prayers for healing, went to mass. I was a leader for two years (...) and it was only when I lost my sight that I handed the group over to a friend, because I was rebellious, that I was such a believer and God was giving me a beating and I simply quit the meetings. (...) At first it was a time of hope, when I went to those masses and hoped that something would change, and later, when nothing helped, I said goodbye to God.

Crises of this kind are characteristic of spiritual pilgrims, who may experience moments of breakdown as a result of disillusionment with both their faith as a whole and with certain demonstrably ineffective practices. S2’s attitude was closest to spiritual settlement:

A lady called at the rectory and asked when the healing mass was. I answered her that every mass is a healing mass. Half joking, half serious, because that is also the truth – You get what you pray for, right? But quite often a person needs some such extras. People who do not impose themselves on God, attend church on Christmas, Easter or Ash Wednesday, because something is happening, there are some extras, there is some action. But with these masses, everyone has their own piety, their own inner need. If someone needs to go to masses with prayers for healing, so help me God. I know there is a fashion for this, but my private opinion is that I as I should go to such a mass and pray for God's will in all this, in healing. If the Lord God sees fit to heal my eyes – so help me God, but if he heals my spirit – so help me God. Because we request the Lord God what we think He should provide according to me, not what He thinks.

K2, on the other hand, reported experiencing healing from a serious hearing impairment, which occurred during intercessory prayer at the support group during the Alpha course:

It's an interesting story in general, because before just going on the course I had a very big hearing loss. I had an accident (...) I overestimated my strength and something in my head failed, because I am a neurologically impaired person and I had a very big loss of hearing. I was a bit apprehensive going there, and at dinner a group from the community just prayed over me and my hearing loss went back a lot.

These types of situations were most often treated by respondents as tangible signs of God's activity and, when linked to the community, offered the possibility of strong bonds based on awakening a sense of belonging. However, this results in the danger of communities being treated instrumentally in relation to specific expectations which thus, if not met, will lead to individuals leaving the community. Moreover, this demonstrates the significance of the community in this case. It is worth considering that it is not individual religious practices that are referred to, but group prayers. This may suggest that in the lives of spiritual settlers and pilgrims, the religious community plays a key role. The close relationship between people with disabilities and non-disabled people has a positive impact on the desire for empowerment and showing initiative, as exemplified by K2, A2 and S2’s activities such as being involved in a music band, running a Sunday school or organising trips with people with disabilities.

### Spiritual Wanderers

Spiritual wanderers; on the surface, may seem similar to spiritual pilgrims. However, these wanderers do not have an end goal, which can result in them joining many different communities in the short-term, sometimes several at the same time. These people thus often choose a group because they need something from it. Such instrumental approaches can relate to a variety of issues however: for some, it is the expectation of signs of God’s activity, while for others it relates to access to facilities, such as a musical ensemble in R1’s case:

I only get involved because I don't want to be stuck in a musical malaise. There is no place in Ciechocinek (small town in Poland) where you can really get involved in music.

This treatment of the community as a tool to satisfy one's own needs, in an instrumental way, differs from the attitudes of both spiritual settlers and pilgrims. Spiritual progress is not the primary goal for such wanderers, and they feel no obligation to be constantly on the road. Consequently, they may temporarily belong to a particular community which they later abandon, either moving on to another group or ceasing their search. This was the case for A3, who, when asked about her searches for a religious group she would like to get involved in, replied:

I haven’t looked around yet, more like something at my place, in my location, because I live somewhere else now (...). And I am also from outside of Warsaw, so I don't know if I will be in Warsaw permanently or not, for now I am. I am in different places, so it’s also like, once here, once there.

This type of spiritual transience does not contribute to strengthening the bonds between members of a community, and this can hinder intra-group engagement and spiritual progress. R1 talked about keeping his distance in terms of the “practical role of community”:

God is what I seek in my own way, but more musical possibilities to be honest. I keep God at a distance perhaps, so to speak. That is the practical role of this community. I am interested in sound engineering, so recently even when I was at the anniversary of the death of Father John I met a man who was involved in the sound system there and, so to speak, two sound engineers met and we spent half an hour discussing what kind of sound system was used, how it was assembled.

Spiritual wanderers did not particularly focus on the relationships with other members of the community in their narratives. They sometimes pointed to a common element, such as a passion for music, but it was a particular slice of reality, rather than the community as a whole, that they were more interested in. A focus on needs as dominating community engagement was evident in this group, though this should not be interpreted in a pejorative manner. No specific attitudes necessarily have a lifelong character and their presentation depends both on the respondent’s stage of life, their current spiritual development, and many other factors; investigating the interactions of these factors could thus become a starting point for the verification or generation of new theories in this area.

### Strengths and Limitations

This study presents several strengths as well as several weaknesses which should be noted when analysing its results. It involved 10 respondents and is therefore exploratory in nature and a preliminary stage in the scientific process. As such, its results cannot be applied to people with visual impairment in general. This is particularly so, because consent to participate in the study was given by a small proportion of those asked. It can be assumed that the willingness to participate in the study was only expressed by people who, despite their disability, are characterised by relatively well-developed social competences. It can therefore be assumed that the ways in which people with lower social competences find themselves in the community may be different. Despite the limitations, the results obtained may provide a starting point for further reflection and research, and in particular for the development of tools with which to study a larger population of visually impaired people. It is worth noting, however, that this is a topic rarely addressed in the literature, so the results presented in this text may provide grounds for further scientific reflections related to the participation of people with visual impairment in the religious community. This can be based on both a qualitative and quantitative strategy, which allows us to take a broader look at the topic by exploring further research fields related to the levels of social inclusion and the community as a space for spiritual development. This is important not only for individual religious communities and their members, but also for the rest of society.

## Conclusion

Numerous studies have been performed on the relationship between religiosity or spirituality and human health that have recognised that spiritual well-being plays a very important role in helping people to cope with illness and disability, thus protecting people from falling into despair (Seeman et al., 2003; Thoresen, 1999). Within such global discourse, there has been an extensive focus on quality of life and religious practices; however, setting this in a community context is very important. This social dimension offers context associated with transcending oneself in favour of caring for something that, from a materialist and formalistic perspective does not directly belong to the individual, but which, because of the value attributed to it, is seen to act as such; it can thus act as the starting point for further research in this area.

Persons with visual impairment who are members of religious communities can be divided by the existence of three types of attitudes into spiritual settlers, pilgrims, and wanderers. These are characterised by different approaches to community in terms of presence, belonging, and commitment, which in turn leads to differing attitudes towards the common good and the resulting actions taken to ensure this. Thus, the effect of the respondents adopting a certain attitude emerges in the different ways they treat their religious community. This may occur due to the implementation of one of the positive mechanisms that allows religious coping within difficult situations, trusting those perceived to be connected to God and the Church (Pargament, 1997), a process emphasised in religious communities. The most important determinant of this, however, is not formal membership nor physical ability, but rather the attitude towards the common good and the activities undertaken for the benefit of the community.

Despite the fact that the respondents represented both Catholic and Protestant denominations, their attitudes towards the religious community were not significantly different. A distinctive feature of Protestant churches and their services is the close bond shared among the people who attend them. This feature is connected, among other things, with additional meetings and a greater familiarity with other believers than is the case in a Catholic Church. However, in the case of Catholic communities, whose members meet not only at common services, but these bonds were also evident in other instances, which indicates the multidimensional roles played by religious communities in the lives of people with visual impairments (Maciejewska, 2021). Indeed, these roles relate both to individuals’ self-esteem and sense of usefulness, and to their taking on new responsibilities and building social bonds.

Thus, answering the key question of this article: What is the nature of the involvement of persons with visual impairment in religious communities? It has to be said that among people with disabilities who are involved in religious communities, we can distinguish those who treat the community instrumentally and seek their benefits associated with membership, as well as such who get involved for the sake of other people, they take responsibility for the community. Spiritual wanderers who are not characterised by a strong commitment to the religious community and the establishment of close ties with its members often treat it in an object-like manner. Consequently, the actions they take are primarily intended to benefit them. In contrast, there are spiritual settlers, who treat the religious community in a subjective manner, take care of it and take responsibility for it. These activities concern different areas—some of them require leadership skills (e.g. running a Sunday school), others group work skills (e.g. in a music band), and others organisational skills (e.g. organising trips for people with disabilities). Regardless, persons with visual impairment need support from non-disabled people also in religious spaces. This is an important task because of the need for social inclusion. Thus, keeping in mind the aim of the research conducted, an attempt was made to identify factors that may be important in the process of including people with disabilities in religious communities. These include giving persons with visual impairment the opportunity to take on new challenges, to take initiative, to involve them in open communities where non-disabled people are also active, and to use their aptitudes and identify their talents to enable them to be truly involved rather than just be or belong.

Thus, keeping in mind the religious dimension of human functioning and the holistic approach to human development (Page et al., 2020), it is important to remember to develop the initiative and self-esteem of individual community members who, regardless of their level of ability, want to participate in the common good. For this inclusion, in addition to infrastructural solutions, what is needed above all is social relationships that provide a sense of security and allow people to take initiative and undertake diverse activities for self-development, the subjective treatment of important reference groups and the common good regardless of their level of ability. It is the practices of communities, i.e. among others, religious communities—also at the lowest, parish levels—that play a special role in the context of the aforementioned process of social inclusion, where, although it is important to recognise the specific needs of people with disabilities, it is above all to enable integral development at all levels (Domagała-Zyśk, 2008).

## References

[CR1] Abarghouei M, Sorbi MH, Abarghouei MR, Bidaki R (2017). The relationship between religious coping strategies and happiness with meaning in life in blind people. Global Journal of Health Science.

[CR2] Borowska-Beszta B (2021). Artefakty inkluzji kulturowej osób z niepełnosprawnością fizyczną w badaniu dostępności zabytkowych sanktuariów. Raport z fotoetnografii obiektu. Przegląd Badań Edukacyjnych.

[CR3] Brennan M, Horowitz A, Reinhardt JP, Cimarolli V, Benn D, Leonard R (2001). In their own words: Strategies developed by visually impaired alders to cope with vision loss. Journal of Gerontology and Social Work.

[CR4] Brennan M, MacMillan T (2008). Spirituality, religiousness and achievement of vision rehabilitation goals among middle-age and older adults. Journal of Religion, Spirituality & Aging.

[CR5] Campbell JD, Yoon DP, Johnstone B (2010). Determining relationships between physical health and spiritual experience, religious practices and congregational support in a heterogeneous medical sample. Journal of Religion and Health.

[CR6] Carter E, Tuttle M, Spann E, Ling Ch, Jones T (2022). Addressing accessibility within the church: Perspectives of people with disabilities. Journal of Religion and Health.

[CR7] Chlan KM, Zebracki K, Vogel LC (2011). Spirituality and life satisfaction in adults with pediatric-onset spinal cord injury. Spinal Cord.

[CR8] Chudy W, Mazanka P (2001). Człowiek niepełnosprawny w świetle filozofii. Filozofia i teologia w życiu człowieka.

[CR9] Cudowska A, Nikitorowicz J, Muszyńska J, Sobecki M (2009). Wspólnota w kulturze indywidualizmu. Wspólnoty z perspektywy edukacji międzykulturowej.

[CR10] Domagała-Zyśk E (2008). Integral development of students with special educational needs in inclusive education from a personalistic perspective. Paedagogia Christiana.

[CR11] Domagała-Zyśk E (2017). Christian context of disability and special education nowadays. Journal for Perspectives of Economic Political and Social Integration.

[CR12] Duda, M. (2014). Trudny dialog osób niepełnosprawnych w kontekście poszanowania ich godności. In M. Duda, & A. Świerczek (Eds.). *Dialog powinnością człowieka wierzącego* Wydawnictwo św. Stanisława BM.

[CR13] Frankl, V. E. (2014). *Człowiek w poszukiwaniu sensu*. Wydawnictwo Czarna Owca.

[CR14] Giaquinto S, Spiridigliozzi C, Caracciolo B (2007). Can faith protect from emotional distress after stroke. Stroke.

[CR15] Głaz, S. (2006). *Sens życia a religia*. Fundacja Humaniora.

[CR16] Glover-Graf NM, Marini I, Baker J, Buck T (2007). Religious and spiritual beliefs and practices of persons with chronic pain. Rehabilitation Counseling Bulletin.

[CR17] Gomes, K. C. (2002). *Relações de família: A complexidade dos grupos multi familiares do Programa Rede de Apoio e Proteção à Família*. Ano de Obtenção.

[CR18] Horowski J (2019). Wspólnota przestrzenią odkrywania sensu życia. Implikacje teorii socjologicznej Margaret S. Archer.. Teologia i Moralność.

[CR19] Instytut Statystyki Kościoła Katolickiego. (2020). *Annuarium Statisticum Ecclesiae in Polonia. Dane za 2019 r.* https://www.iskk.pl/images/stories/Instytut/dokumenty/Annuarium_Statisticum_DANE_2019_FINAL.pdf.

[CR20] Janocha, W. (2011). *Religijność osób niepełnosprawnych i ich rodzin. Studium socjologiczno-pastoralne*. Wydawnictwo KUL.

[CR21] Johnstone B, Franklin KL, Yoon DP, Burris J, Shigaki Ch (2008). Relationships among religiousness, spirituality and health for individuals with stroke. Journal of Clinical Psychology in Medical Settings.

[CR22] Johnstone B, Glass BA, Oliver RE (2007). Religion and disability: Clinical, research and training considerations for rehabilitation professionals. Disability and Rehabilitation.

[CR23] Koenig HG, King DE, Carson VB (2012). Handbook of religion and health.

[CR24] Krąpiec M, Maryniarczyk A (2001). Dobro wspólne. Powszechna encyklopedia filozofii.

[CR25] Krause N, Hayward RD (2012). Religion, meaning in life, and change in physical functioning during late adulthood. Journal of Adult Development.

[CR26] Lipiec, D. (2011). *Duszpasterstwo niewidomych i słabowidzących w Polsce. Studium teologiczno-pastoralne*. Wydawnictwo KUL.

[CR27] Maciejewska M (2021). Niewidomi we wspólnocie religijnej. Potrzeby i trudności [The blind in the religious community. needs and difficulties]. Roczniki Pedagogiczne.

[CR28] Marini I, Glover-Gra NM (2011). Religiosity and spirituality among persons with spinal cord injury: Attitudes, beliefs and practices. Rehabilitation Counseling Bulletin.

[CR29] Mikołajewska, B. (1999). *Zjawisko wspólnoty*. The Lintons’ Video Press.

[CR30] Page RL, Peltzer JN, Burdette AM, Hill TD (2020). Religiosity and health a holistic biopsychosocial perspective. Journal of Holistic Nursing.

[CR31] Pargament KI (1997). The Psychology of religion and coping.

[CR32] Ryan RM, Deci EL, Deci EL, Ryan RM (2004). Overview of self-determination theory: An organismic dialectical perspective. Handbook of self-determination research.

[CR33] Seeman TE, Dubin LF, Seeman M (2003). Religiosity/spirituality and health. A critical review of the evidence for biological pathways. American Psychologist.

[CR34] Szabała B (2021). Religijność dorosłych osób słabowidzących w kontekście struktury i uwarunkowań. Niepełnosprawność–dyskursy Pedagogiki Specjalnej.

[CR35] Thoresen CE (1999). Spirituality and health: Is there a relationship?. Journal of Health Psychology.

[CR36] Tönnies F (2012). Community and society.

[CR37] Von Hildebrand, D. (1930). *Metaphysik der Gemeinschaft: Untersuchungen über Wesen und Wert der Gemeinschaft*. Haas & Grabherr.

[CR38] Waldorn-Perrine B, Rapport LJ, Hanks RA, Lumley M, Meachen SJ, Hubbarth P (2011). Religion and spirituality in rehabilitation outcomes among individuals with traumatic brain injury. Rehabilitation Psychology.

[CR39] Wuthnow R, Fenn RK (2003). Spirituality. The blackwell companion to sociology of religion.

[CR40] Yampolsky MA, Wittich W, Webb G, Overbury O (2008). The role of spirituality in coping with visual impairment. Journal of Visual Impairment & Blindness.

[CR41] Zaffalon-Peter M, Jabbar-Peter PF, Catapan AH (2015). Belonging: Concept, meaning and commitment. US-China Education Review B.

